# Evolution of Rapid Development in Spadefoot Toads Is Unrelated to Arid Environments

**DOI:** 10.1371/journal.pone.0096637

**Published:** 2014-05-06

**Authors:** Cen Zeng, Ivan Gomez-Mestre, John J. Wiens

**Affiliations:** 1 Department of Biology II, University of Munich, Munich, Germany; 2 Ecology, Evolution, and Development Group, Doñana Biological Station, Consejo Superior de Investigaciones Científicas, Seville, Spain; 3 Department of Ecology and Evolutionary Biology, University of Arizona, Tucson, Arizona, United States of America; Laboratoire Arago, France

## Abstract

The extent to which species' life histories evolve to match climatic conditions is a critical question in evolutionary biology and ecology and as human activities rapidly modify global climate. GIS-based climatic data offer new opportunities to rigorously test this question. Superficially, the spadefoot toads of North America (Scaphiopodidae) seem to offer a classic example of adaptive life-history evolution: some species occur in extremely dry deserts and have evolved the shortest aquatic larval periods known among anurans. However, the relationships between the climatic conditions where spadefoots occur and the relevant life-history traits have not been explicitly tested. Here, we analyzed these relationships using GIS-based climatic data, published life-history data, and a time-calibrated phylogeny for pelobatoid frogs. Surprisingly, we find no significant relationships between life-history variables and precipitation or aridity levels where these species occur. Instead, rapid development in pelobatoids is strongly related to their small genome sizes and to phylogeny.

## Introduction

Variation in climate over space and time may be an important factor driving evolutionary changes in life-history among and within species [1], [2]. Studying this relationship between climate and life-history evolution has taken on new urgency as climate has begun to change rapidly and impact natural populations [3]–[6]. The combination of GIS-based climatic data and phylogenetic comparative methods now provides the opportunity to rigorously test hypotheses relating climatic variation to life-history variation among species (e.g. [7]–[9]). However, to our knowledge, no studies have used this approach to test for the environmental correlates of developmental rates.

The spadefoot toads of North America (Scaphiopodidae) seem to offer a classic example of adaptation in life-history variables to extreme climatic conditions. Although frog species richness is strongly correlated with mesic environments [10], scaphiopodid spadefoot toads occur in all the desert regions of North America, and some species occur in the driest regions within these deserts [11]. Seemingly in association with this environment, they have extremely short larval periods [12], and one species (*Scaphipus couchii*) that occurs in the driest regions of North America is thought to have the shortest aquatic larval period among all the >6,000 species of frogs [13], [14]. Spadefoot toads spend much of the year underground but are active on the surface during rainy periods (in summer for most species), when they emerge to forage and breed in temporary pools filled by rain [11]. These pools often dry quickly, and spadefoot toads appear to have evolved very rapid development to allow their eggs to develop and hatch, and the aquatic larvae to grow and metamorphose, before these pools dry [15]–[18]. It seems intuitive that regions with lower precipitation would tend to have smaller temporary pools that dry out more quickly (given their smaller size), which could lead to a strong relationship between macro-climatic precipitation levels where species occur and their rates of larval development. Many previous authors have noted that rapid pond drying leads to high tadpole mortality and that development is rapid in desert-dwelling tadpoles, and that rapid development may therefore be an adaptation allowing survival in these climates (e.g. [13]–[15], [19]). However, the seven species of scaphiopodid spadefoot toads occur in a variety of habitats across North America, from arid deserts to mesic temperate forests [11], [20]. No previous study has explicitly tested whether their rapid developmental rates are actually related to occurrence in more arid environments using explicit climatic data and phylogenetic comparative methods. For example, Buchholz & Hayes [18] suggested that developmental traits in pelobatoids were related to phylogeny rather than habitat, but without data on climate or use of phylogeny-based tests.

Here, we test the relationships between environmental conditions and life-history traits among species of pelobatoid frogs. Based on current classifications [21], [22], the pelobatoid frogs include the scaphiopodids (North American spadefoot toads) and the pelobatids (Eurasian spadefoot toads) and two other families that interdigitate among the two clades of spadefoot toads (the Eurasian Pelodytidae and Asian Megophryidae). We synthesize existing data in the literature on relevant developmental traits in these species, specifically larval period and hatching time. We also include available data on genome size, given that small genome size is associated with rapid development in many organisms [23], although this has not been tested in a phylogenetic context in frogs (to our knowledge). We then test how these traits are related to environmental conditions where these species occur. We obtain GIS-based climatic data from georeferenced localities for these species, focusing on variables most likely to determine the water available from rainfall for larval development (annual precipitation, precipitation of the wettest quarter, precipitation seasonality, aridity). We then analyze these data in the context of a time-calibrated phylogeny. Specifically, we test whether pelobatoid species occurring in drier environments have shorter hatching times and larval periods, and whether shorter hatching times and larval periods are related to smaller genome sizes. We also test how life-history traits, genome size, and climatic distributions are related to the phylogeny. Our study also generates a well-supported, multi-locus, time-calibrated Bayesian phylogeny for scaphiopodids, providing a resource for comparative studies on this model system in evolution, ecology, development, and behaviour [14], [17], [24]–[29]. Our results show that evolution of rapid development in spadefoot toads is not related to occurrence in drier climates, but that there are significant relationships between developmental rates and genome size.

## Materials and Methods

### Life-history data

We searched the literature for data on relevant life-history variables for all pelobatoid species (Appendix S1), starting from the summary provided in Gomez-Mestre & Buchholz [24]. Whenever possible, we used only data measured in the field under natural conditions. For those few species lacking field-based data for a specific variable, data from the lab were used instead. We obtained data on larval period (from hatching of the eggs to approximately Gosner stage 42) and hatching time (from egg deposition to hatching). For each variable, when multiple records were available for one species, we obtained the maximum and minimum values from the available records and calculated the midpoint. Data were available for most scaphiopodid, pelobatid, and pelodytid species, but relevant data were available for only two megophryid species. We note that megophryids are geographically and climatically distinct from the other pelobatoid families, occurring primarily in mesic tropical and subtropical areas of Asia [21].

Data on genome sizes were obtained from T. R. Gregory's database (http://www.genomesize.com/). Data were available for only eight pelobatoid species, but these species included two from each genus for all the genera in the Pelobatidae, Pelodytidae, and Scaphiopodidae. Summary life-history data for each species for each variable are provided in Table S1.

### Climatic data

To obtain climatic data for each species, we first obtained georeferenced locality data. We used species distribution data from HerpNET (www.herpnet.org) and the Global Biodiversity Information Facility (GBIF; www.gbif.org; Version 1.2.6). HerpNET and GBIF both provide a frequently updated database of museum specimen records. We searched each database for each species, and then combined the records into a set of unique localities for each species. Sample sizes ranged from 3 to 322 localities per species (mean  = 89.1).

Localities for each species were visualized using DIVA-GIS version 7.5.0.0 and compared to species distribution maps from the IUCN Red List of Threatened Species, version 2012.2 [30]. The known distributions of these species are relatively stable and agreed upon by different sources [11], [20], [30]. Localities falling outside the IUCN distribution map were excluded. We also excluded localities with estimated elevations (from WorldClim, see below) that fell outside the range of reported elevations from IUCN.

Using this carefully vetted set of georeferenced localities, we then obtained climatic data from the WorldClim (version 1.3) database [31]. Data are based on averages from weather stations from the years ∼1950–2000 (with spatial interpolation to localities between weather stations), with a spatial resolution of ∼1 km^2^. For each locality, we extracted data on annual precipitation (Bio12), precipitation seasonality (Bio15), and precipitation of the wettest quarter (Bio16). We expect these variables to have the strongest influence on rainfall available for filling temporary ponds for anuran reproduction. Maximum and minimum values across localites within the species range were obtained, as well as the midpoint of these two values, and the mean value for the species averaged across all localities.

We also used a measure of aridity per se, following Oufiero *et al*. [32]. For each species, this was calculated as 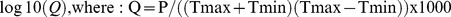
where P is annual precipitation (mm; mean across localities across species range), Tmax is the maximum value of Bio5 (maximum temperature of warmest month) across the species range, and Tmin is the lowest value of Bio6 (minimum temperature of the coldest month). Arid environments have a lower Q [33]. We also used logQ2, in which Tmax is the mean value of Bio10 (mean temperature of warmest quarter) across localities and Tmin is the mean value of Bio11 (mean temperature of coldest quarter), but this gave similar results. A summary of the climatic data for each species is provided in Table S1.

### Time-calibrated phylogeny

We estimated a time-calibrated phylogeny for pelobatoid frogs, since one including all relevant taxa was not available at the time we initiated our study (and one available now has some issues, see below). We first reduced the data matrix compiled by Pyron & Wiens [22] to include only the 16 pelobatoid species for which life-history data were available. However, these data were available for only two megophryid species, and life-history data were available for *Leptobrachium nigrops* but not sequence data. Therefore, rather than exclude this species and genus, we used sequence data from *Leptobrachium chapaense* to represent *L. nigrops* in the tree. We also excluded genes sampled for fewer than 4 included species. The resulting data matrix included 16 species and data from the mitochondrial ribosomal genes 12S and 16S, the mitochondrial protein-coding gene cytochrome *b*, and the nuclear protein-coding genes H3A, RAG1, RHO, SIA, and SLC8A3 (total length of combined alignments  = 9,355 base pairs). Data were not available for all 8 genes for all 16 species, and some species were therefore missing data for some genes. However, both simulations and empirical studies suggest that including some missing data need not lead to inaccurate estimates of phylogeny, especially when a large number of characters is sampled overall (review in [34]).

The time-calibrated tree was estimated using the Bayesian uncorrelated lognormal approach in BEAST 1.5.4 [35], [36]. We used the GTR + I + Γ model (following [22]) with 4 rate categories for Γ, and estimated base frequencies. We used a clock model with the uncorrelated, lognormal approach, and an estimated rate. The starting tree was based on a Yule speciation prior.

We initially used two fossil calibration points. For each point, we identified a fossil that represented the oldest taxon that could be confidently assigned to a clade of extant species. A fossil can be used to determine the minimum age of a clade, but the clade can be older than this oldest known fossil. We therefore used a lognormal prior distribution for each calibration point, with an offset equal to the minimum age of the oldest fossil (the youngest age of the oldest stratum in which it is found) and a mean of 5 and a standard deviation of 1 Myr. This combination of mean and standard deviation yields a 95% prior distribution that extends from just slightly older than the minimum age of the fossil to approximately 15 Myr older (an arbitrary but seemingly plausible range), with the highest probability slightly older than the age of the fossil.

We initially used the crown-group age of Pelobatoidea as being at least 50.3 Myr old, given a fossil scaphiopodid (*Scaphiopus guthriei*) from the Wind River formation (lower Eocene Wasatchian 50.3–55.4 Mya) following Rocek & Rage [37]. The 95% interval on the prior is 50.9–66.0 Myr.

We initially treated the crown-group age of Pelobatidae and Megophryidae as being at least 33.9 Myr old, given the fossil *Eopelobates grandis* which appears to be closely related to *Pelobates* [37], [38], from the Chadron formation (33.9–38 Mya). The 95% interval on the prior for Pelobatidae+Megoprhyidae is 34.5–49.6 Myr. This clade may be older if the undescribed “Green River pelobatid” can be assigned to it (Wasatchian, 50.3–5.4 Mya; [37]). Further, the most recent common ancestor of Pelodytidae, Pelobatidae, and Megophryidae is also at least 33.9 Mya, given fossil *Pelodytes* from the late Eocene (37.2–33.9 Mya; [37]), but we did not use this fossil calibration (given that the pelobatid + megophryid calibration ensures that this clade is at least this old).

Initial analyses yielded family-level topologies that did not match those of Pyron & Wiens [22], most likely due to the lack of outgroups. Given that these family-level relationships are generally well supported when outgroups are included (e.g. [22], [39]–[41]), we constrained these relationships. Specifically we constrained the clade: Pelodytidae+Pelobatidae+Megophryidae. Given that the pelobatid+megophryid clade is a fossil constraint, these two constraints enforce the Pyron & Wiens [22] topology for families. Importantly, the same set of family-level relationships is also found in other previous analyses of pelobatoid relationships, including those based on mitochondrial data only [39], nuclear data only [40], and combined nuclear and mitochondrial data [41].

We performed two independent runs each with 50,000,000 generations sampled every 1,000 generations. We used the maximum clade credibility trees with mean node heights. The first 10% of generations sampled were discarded as burn-in using TreeAnnotator version 1.5.4 and viewed using FigTree version 1.3.1 [35]. We confirmed that the two independent runs gave effective sample sizes (ESS) greater than 200 for the likelihood and selected clade ages, and that they converged on similar topologies and divergence dates. Trees from the two analyses were combined to yield a majority-rule consensus tree with mean branch lengths.

This initial analysis yielded estimated ages for Pelobatoidea and the family-level clades within it that were considerably younger than those estimated in previous studies (e.g. [40]–[42]). For example, the pelobatoid crown group was 53 Myr old in this tree, and ∼130, 170 and 150 Myr old (respectively) in these previous studies. Therefore, we reran the analyses as above, but making two changes. First, for the crown age of Pelobatoidea, we used a normal prior distribution with a mean age of 150 Mya and a standard deviation of 10 Myr (95% prior interval: 133.6–166.4). This prior interval roughly corresponds to the range of estimated ages in previous studies. Second, we used the fossil calibration for *Scaphiopus guthrei* for the crown-group age of Scaphiopodidae, rather than the stem-group age (this choice is less conservative about the placement of this fossil but more in line with previous age estimates).

**Figure 1:**
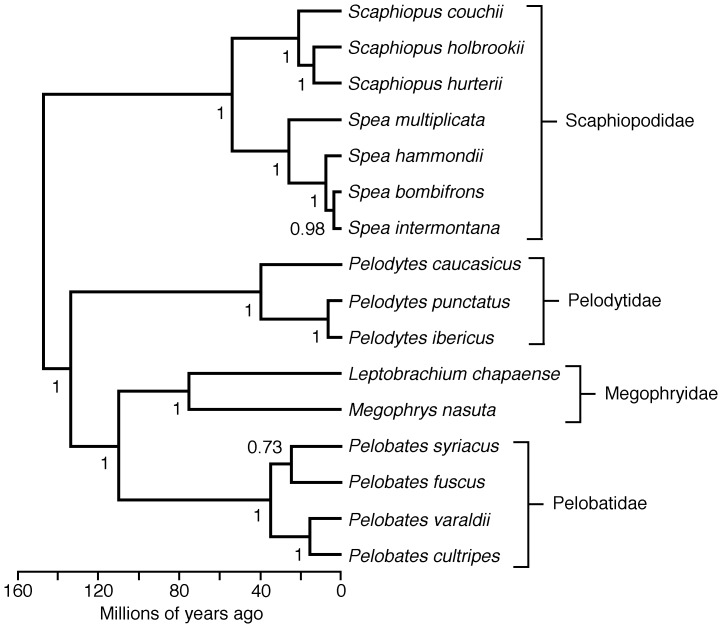
Time-calibrated phylogeny of pelobatoid frogs used in comparative analyses. Numbers at nodes indicate Bayesian posterior probabilities of clades.

This second set of results gave an identical topology and similar relative branch lengths to the first analysis, but with absolute branch lengths (ages) similar to those estimated in previous studies. We used this second BEAST tree for our phylogenetic comparative analyses. The topology was very strongly supported, with only one node with a posterior probability <0.95 (Fig. 1). Therefore, we did not incorporate uncertainty in the phylogeny into our comparative analyses. This topology is available in nexus/newick format in Appendix S2.

Note that another study has recently estimated a large-scale time-calibrated tree for amphibians, including pelobatoids [43]. However, given the large number of taxa included in that study, the use of a somewhat suboptimal method for estimating divergence dates was necessary (penalized likelihood; [44]). Furthermore, that study [43] relied entirely on secondary calibration points (from [42]). Therefore, we prefer our estimate of divergence dates. Nevertheless, these estimates are actually quite similar (and similar to estimates in other recent studies [7], [40]–[42]), and are based on nearly identical molecular datasets [22].

### Phylogenetic comparative analysis

We tested the relationship between pairs of variables using phylogenetic generalized least squares (PGLS; [45]) as implemented in the R package *caper*, version 0.5 [46]. Prior to conducting these analyses, we found the best-fitting evolutionary model for each variable using the R packages *ape* [47] and *geiger* [48]. We compared the fit of the models using the estimated likelihood and Akaike information criterion (AIC), with an AIC difference of 4 or greater indicating support for alternative models [49]. We compared the Brownian motion (BM; perfect fit of a character to the phylogeny), Ornstein-Uhlenbeck (OU; equivalent to stabilizing selection around a single optimum), and estimated lambda (level of phylogenetic signal is estimated) models (see Table S2). We found that in most cases, model fit was similar between the OU and lambda models (AIC difference <4), with the exception of two climatic variables for which OU was strongly favored (annual precipitation, wettest quarter precipitation). We therefore used the lambda model in PGLS, given that this model was either favored and/or alternate models were not. We also performed a set of analyses using the OU model. Specifically, we repeated the PGLS analyses after using *geiger* to transform the tree based on the OU model and the estimated value of alpha. To estimate alpha, the selected variable was fitted to the OU model 10 times and the alpha with the minimum deviance (−2 * log-likelihood) was applied in the transformation [50]. PGLS results were generally similar using the lambda and OU models, and we present the results using the lambda model as our primary results. We present the OU results as supplementary information (Table S4).

We used PGLS to test the following specific hypotheses. (1) We predicted that overall hatching times and larval periods of species will be related to their mean values for climatic variables (annual precipitation, precipitation of the wettest quarter, aridity), assuming that species reduce their hatching times and larval period to allow them to metamorphose before temporary breeding ponds dry in more arid climates. For these analyses, we summarized variation in hatching times and larval periods within species based on midpoint values (midpoint between lowest and highest values within species). (2) We predicted that the minimum (shortest) hatching times and larval periods within species will be related to the lowest values for annual precipitation related variables across the range of each species. This second set of analyses was intended to address the possibility that overall species values might not reflect variation within species, and that we expect shorter larval periods and hatching times in parts of the species range with lower rainfall. (3) We predicted that hatching times and larval period would be related, assuming that species occurring in drier environments will evolve to minimize both simultaneously. We examined both midpoint and minimum values for these variables within species. (4) We predicted that genome sizes would be smaller in species with more rapid development (shorter hatching times and larval periods).

We acknowledge that the methods described above could lead to a potential mismatch between developmental traits and climatic variables for specific localities (e.g. for a given species, the locality with the shortest recorded larval period may not correspond to the driest locality where the species occurs). Therefore, we performed an additional analysis in which both developmental and climatic values for each species were based on a single locality with the shortest recorded field-based larval period for that species (focusing on annual precipitation). However, there were some issues in this analysis. First, the data on larval period for four species could not be traced to specific localities (i.e. *Pelodytes caucasica, Pelobates syriacus, Pelobates varaldii, Megophrys nasuta*). For these species, we used data on the minimum recorded larval period (from the field) and the lowest annual precipitation across sampled localities. In several other species, it was not possible to trace the shortest recorded larval period known for that species to a specific locality. In these cases, we simply used the shortest larval period for a given species that could be traced to a specific locality. Although these larval periods were sometimes longer than the shortest larval periods recorded for that species, they may also be more reliable, and should provide a strong overall test of how climate and larval period are related. The data and references used are summarized in Appendix S3 and Table S3.

We also tested each variable for phylogenetic signal using lambda [51]. Given that the phylogenetic results were very strongly supported (Fig. 1) and similar to previous estimates (see above), we did not test the robustness of the results of the comparative analyses to alternative trees.

## Results

**Figure 2:**
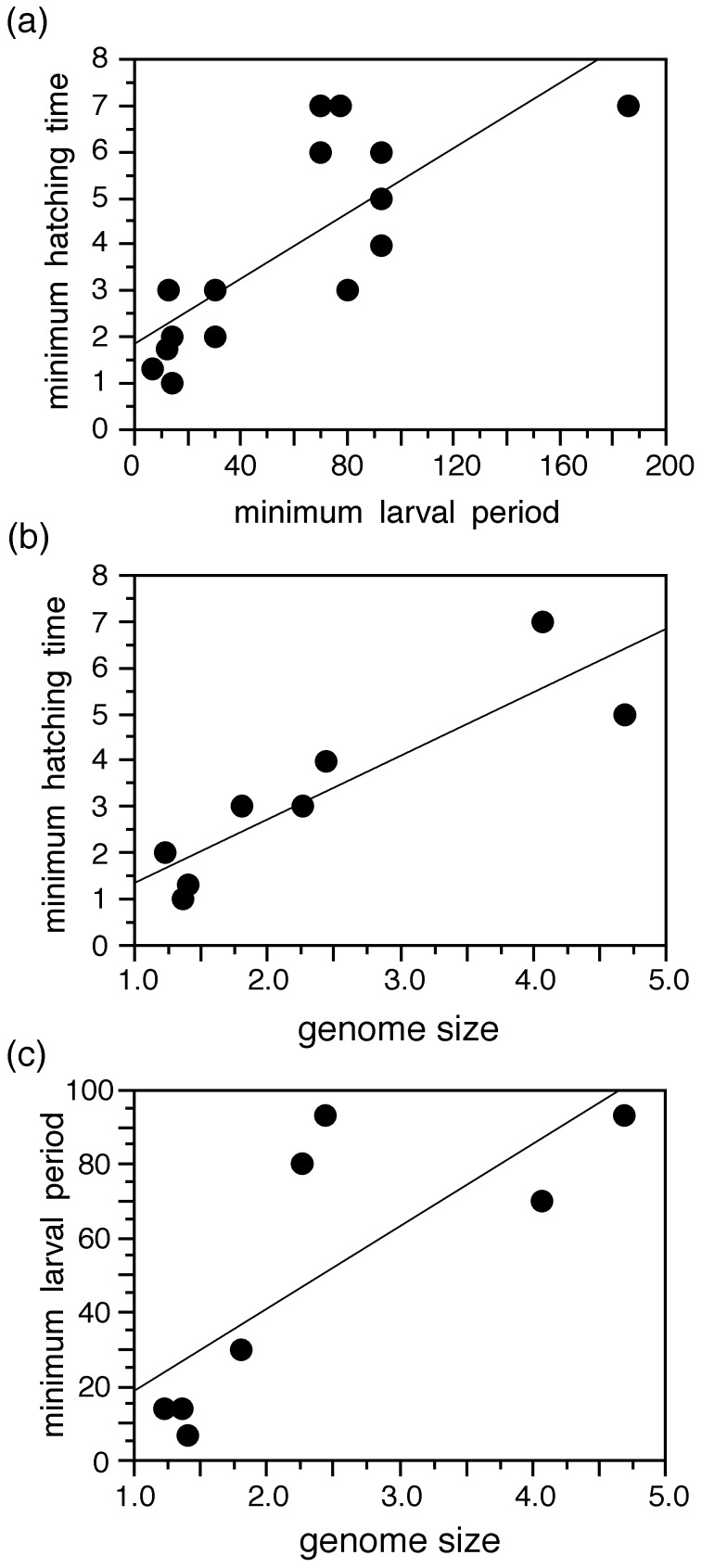
Relationships between selected life-history variables and genome size (from among the significant relationships in Table 1). For ease of visualization, we plot the raw data and standard regression lines (see Table 1 for PGLS results). Larval periods and hatching times are given in days; genome sizes are given as C-values in picograms.

Surprisingly, we find no relationship between larval period and the climatic variables nor between hatching time and the climatic variables (Table 1), using either mean/midpoint or minimum values. There is also no relationship when using data on larval period and climate from specific localities (Table 1). There is no significant relationship between midpoint hatching time and midpoint larval period, but there is a strong relationship using the minimum values within species for both variables (Fig 2a; Table 1). There are strong relationships between genome size and minimum hatching times and between genome size and minimum larval period (Fig. 2b,c), but not between genome size and mipdoint hatching times. Results are generally similar using the OU model (Table S4), especially in the non-significant relationships between climate and developmental rates. However, the relationships between minimum hatching time and minimum larval period and minimum hatching time and minimum genome size are no longer significant under the OU model (although the relationship between midpoint hatching time and larval period is), but OU is not the best fitting model for any of these variables, and so these results should not be preferred to our main results using the lambda model. Most variables (Table 2) show significant but not perfect phylogenetic signal (lambda  = 0.4–0.6), except for precipitation seasonality (lamba <0.01) and genome size (lambda >0.95).

**Table 1:** Relationships between climatic and life-history variables in pelobatoid frogs using phylogenetic generalized least squares (PGLS).

Variables	*R^2^*	*P*-value
midpoint larval period ∼ mean annual precipitation	0.0180	0.7768
midpoint larval period ∼ mean precip. wettest quarter	0.0275	0.6802
midpoint larval period ∼ mean precip. seasonality	0.0002	0.9964
midpoint larval period ∼ aridity (logQ)	0.0147	0.8141
midpoint hatching time ∼ mean annual precipitation	0.1016	0.2659
midpoint hatching time ∼ mean precip. wettest quarter	0.0559	0.4833
midpoint hatching time ∼ mean precip. seasonality	0.1564	0.1288
midpoint hatching time ∼ aridity (logQ)	0.0949	0.2903
minimum larval period ∼ min. annual precipitation	0.0026	0.9640
minimum hatching time ∼ min. annual precipitation	0.0373	0.6158
larval period ∼ annual precipitation (specific localities)	0.0063	0.9160
midpoint hatching time ∼ midpoint larval period	0.0987	0.2760
**minimum hatching time ∼ minimum larval period**	**0.5981**	**0.0001**
**midpoint larval period ∼ genome size**	**0.6245**	**0.0124**
**minimum larval period ∼ genome size**	**0.6566**	**0.0089**
midpoint hatching time ∼ genome size	0.3159	0.1405
**minimum hatching time ∼ genome size**	**0.7860**	**0.0017**

Significant relationships (*P*<0.05) are boldfaced. Median larval periods and median hatching times refers to the midpoint between the highest and lowest values reported for a species (Appendix S1). Minimum refers to the lowest value. For climatic variables, mean refers to the mean among localities for a species, and min. the lowest value among localities within a species.

**Table 2:** Estimation of phylogenetic signal in the traits analyzed here, based on fit to a Brownian motion model of trait evolution using Pagel's [51] lambda.

Variable	Lambda
Mean annual precipitation	0.6426
Mean precip. wettest quarter	0.6631
Mean precip. seasonality	3.88E-07
Aridity (logQ)	0.7264
Minimum annual precipitation	0.5061
Midpoint larval period	0.7435
Midpoint hatching time	0.4605
Genome size	0.9627

Lambda varies from 0 to 1, with higher values indicating stronger phylogenetic signal.

## Discussion

In this study, we use explicit GIS-based climatic data and phylogenetic comparative methods to test the hypothesis that short developmental times in spadefoot toads are associated with occurrence in more arid environments. Surprisingly, we find no relationship between developmental rates (larval period and hatching times) and climate in the geographic areas where these species occur. Instead, we find strong relationships between our two measures of developmental rates (Table 1), between developmental rates and genome sizes (Table 1), and between phylogeny and developmental rates, genome size, and most climatic variables (Table 2). Our conclusions about developmental rates and climate are largely consistent with those of Buchholz & Hayes [18], but are based on explicit statistical analyses of phylogeny and climate.

Why do we find no relationship between climate and developmental rates? The first question to address is whether the absence of this relationship is real or an artifact of our methods or data. We think that the most important source of error in our study is that our between-species analyses require reducing all variation among populations within a species to a single value for that species, and analyzing only values among species. Thus, it might be that within-species variability obscures between-species patterns. For example, a strong relationship between developmental rate and climate may only arise in dry parts of species ranges, and might be obscured by including life-history and climatic data from other parts of the species range. However, we still find no relationship when using minimum values for larval period and hatching time and the driest values for climatic variables across the species range, instead of midpoints and means (although we acknowledge that the localities for climate and life-history in these latter analyses are not precisely matched). In addition, when we do match data on larval period and climate for a specific locality for each of several species, we again find no relationship. Furthermore, we do find significant relationships between minimum larval periods and minimum hatching times, suggesting that significant relationships between life-history variables can be captured using our data and methods.

We argue that the lack of a strong relationship between climate and life-history instead reflects real patterns that are inconsistent with aridity and short development times being closely related. For example, inspecting the raw species data (Table S1), there are dramatic differences in life history between scaphiopodids (short development times) and pelobatids and pelodytids (longer developmental times), despite the overlapping distributions of climatic variables among these families. Furthermore, two of the three species of *Scaphiopus* (*S. holbrookii, S. hurterii*) occur in relatively mesic environments but nevertheless have relatively fast developmental times, as does the more arid-dwelling *S. couchii*. There are also relatively long development times in species that occur in relatively arid environments, such as *Spea intermontana* and *Spea hammondii*. In summary, these patterns help explain why no significant relationship between dry climates and rapid development was observed.

We note that these two main explanations for the lack of relationship between climate and life history are not mutually exclusive. Specifically, there are some patterns among pelobatoid species that are clearly inconsistent with a tight relationship between developmental times and climate. Nevertheless, there may still be important within-species variation in developmental times and climate that may reflect adaptive evolution, but that our between-species approach is relatively insensitive to. Similarly, phenotypic plasticity and local-scale conditions of temporary pools are also known to play an important role in determining developmental rates in spadefoot toads (e.g. [13]–[17], [29]). However, it also appears that variation within species occurs within limited bounds, and that among species variation is much greater than variation within species (Table S1).

We also emphasize that our focus here is on the question of whether short development times are associated with occurrence in regions with dry climate. However, this is not the same as asking whether short larval periods are associated with use of temporary pools of short duration. In fact, species in mesic regions might select pools with short duration (e.g. *Scaphiopus holbrookii*), whereas species in more arid regions with longer development times may utilize more permanent aquatic larval sites (e.g. *Spea hammondii, Spea intermontana*; [17]). Such patterns may help explain the weak relationship between large-scale climate and development time.

In contrast to the comparisons with climate, our results show a strong relationship between genome sizes and developmental rates in pelobatoids (Table 1; Fig. 2b,c). This result is supported despite the limited number of pelobatoid species with available data on genome sizes (8 species vs. 16 species for most other variables). To our knowledge, no previous studies have tested for a relationship between developmental rates and genome size in anurans using phylogenetic comparative methods. However, various non-phylogenetic studies have been performed that suggested a relationship between DNA content and larval period (e.g. [52]–[56]). Further, phylogenetic comparative analyses in salamanders also suggested a relationship between embryonic period (equivalent to hatching time here) and DNA content [57], .

The exact causal relationships between genome size and developmental rate in pelobatoids are unclear. One hypothesis is that rapid development is difficult with larger genomes, leading to evolution of smaller genome sizes in rapidly developing species. Gregory [23] suggested that large genome size acts as a constraint on rapid development, but that other factors drive the evolution of developmental rate besides genome size.

Intriguingly, we find that many climatic variables and life-history traits show relatively strong relationships with the phylogeny (based on values of Pagel's [51] lambda; Table 2), but genome size shows the strongest relationship of all (lambda >0.95). We speculate that genome size may act to constrain evolutionary changes in developmental rates among species, and might help underlie the relationship between trait variation and phylogeny seen in traits relating to developmental rate.

In this study, we show that developmental rates in spadefoot toads are not significantly related to occupation of relatively arid environments, despite the observation that some pelobatoid species with very fast rates occur in very dry environments. We show instead that these measures of developmental rates are significantly related to each other, to phylogenetic history, and to genome size. We note that our results do not rule out the possibility of strong relationships between climate and life history among populations within species nor an important role for phenotypic plasticity and local-scale conditions in determining developmental rates within populations.

## Supporting Information

Table S1 **Summary of life-history and climatic data for each species.**
(DOC)

Table S2 **Comparison of three evolutionary models for each climatic and life-history variable.**
(DOC)

Table S3 **Summary of life-history and climatic data for each species using matched localities for climatic and developmental data for 12 of the 16 species (see Appendix S3).**
(DOC)

Table S4 **Relationships between climatic and life-history variables in pelobatoid frogs using phylogenetic generalized least squares (PGLS), utilizing the OU model.**
(DOC)

Appendix S1 **Data on life history variables in pelobatoid frogs and associated literature references.**
(DOC)

Appendix S2 **Time-calibrated phylogeny used in the comparative analyses in nexus/newick format.**
(DOC)

Appendix S3 **Data on larval period matched to specific localities.**
(DOC)
