# Supplementary material for: Evolution of Rapid Development in Spadefoot Toads Is Unrelated to Arid Environments
Source: PLoS One. 2014 May 6;9(5):e96637. doi: 10.1371/journal.pone.0096637 (PMC4011863; doi:10.1371/journal.pone.0096637)
Supplement: Table S1 — Summary of life-history and climatic data for each species. (DOC) [file pone.0096637.s001.doc]

Table S1. Summary of life-history and climatic variables used in the comparative analyses. Time periods are in days, and precipitation is in mm. Genome sizes are given as C-values in picograms.

|  | Minimum larval period | Maximum larval period | Midpoint larval period | Minimum hatching time |
| --- | --- | --- | --- | --- |
| *Pelodytes caucasicus* | 80 | 124 | 102 | 3 |
| *Pelodytes ibericus* | 70 | 70 | 70 | 6 |
| *Pelodytes punctatus* | 93 | 155 | 124 | 4 |
| *Scaphiopus couchii* | 7 | 40 | 23.5 | 1.333 |
| *Scaphiopus holbrookii* | 14 | 60 | 37 | 1 |
| *Scaphiopus hurterii* | 13 | 35 | 24 | 3 |
| *Spea bombifrons* | 14 | 40 | 27 | 2 |
| *Spea hammondii* | 30 | 57 | 43.5 | 3 |
| *Spea intermontana* | 30 | 60 | 45 | 2 |
| *Spea multiplicata* | 12 | 23 | 17.5 | 1.75 |
| *Pelobates cultripes* | 93 | 186 | 139.5 | 6 |
| *Pelobates fuscus* | 93 | 155 | 124 | 5 |
| *Pelobates syriacus* | 70 | 124 | 97 | 7 |
| *Pelobates varaldii* | 186 | 248 | 217 | 7 |
| *Leptobrachium nigrops* | 62 | 93 | 77.5 | - |
| *Megophrys nasuta* | 77.5 | 108.5 | 93 | 7 |

|  | Maximum hatching time | Midpoint hatching time | Genome Size |
| --- | --- | --- | --- |
| *Pelodytes caucasicus* | 4 | 3.5 | 2.27 |
| *Pelodytes ibericus* | 9 | 7.5 | - |
| *Pelodytes punctatus* | 14 | 9 | 2.44 |
| *Scaphiopus couchii* | 1.333 | 1.333 | 1.405 |
| *Scaphiopus holbrookii* | 15 | 8 | 1.37 |
| *Scaphiopus hurterii* | 3 | 3 | - |
| *Spea bombifrons* | 2 | 2 | 1.235 |
| *Spea hammondii* | 4 | 3.5 | 1.805 |
| *Spea intermontana* | 4 | 3 | - |
| *Spea multiplicata* | 2 | 1.875 | - |
| *Pelobates cultripes* | 6 | 6 | - |
| *Pelobates fuscus* | 11 | 8 | 4.695 |
| *Pelobates syriacus* | 7 | 7 | 4.07 |
| *Pelobates varaldii* | 7 | 7 | - |
| *Leptobrachium nigrops* | - | - | - |
| *Megophrys nasuta* | 7 | 7 | - |

|  | Precip. annual (Bio12) mean | Precip. annual (Bio12) maximum | Precip. annual (Bio12) minimum) | Precip. annual (Bio12) midpoint |
| --- | --- | --- | --- | --- |
| *Pelodytes caucasicus* | 697.25 | 764 | 606 | 685.0 |
| *Pelodytes ibericus* | 631.94 | 828 | 427 | 627.5 |
| *Pelodytes punctatus* | 558.14 | 1020 | 411 | 715.5 |
| *Scaphiopus couchii* | 406.82 | 1042 | 73 | 557.5 |
| *Scaphiopus holbrookii* | 1203.19 | 1559 | 950 | 1254.5 |
| *Scaphiopus hurterii* | 977.00 | 1352 | 220 | 786.0 |
| *Spea bombifrons* | 476.35 | 1025 | 113 | 569.0 |
| *Spea hammondii* | 375.45 | 774 | 153 | 463.5 |
| *Spea intermontana* | 288.77 | 765 | 98 | 431.5 |
| *Spea multiplicata* | 489.28 | 1791 | 200 | 995.5 |
| *Pelobates cultripes* | 509.48 | 828 | 361 | 594.5 |
| *Pelobates fuscus* | 615.23 | 858 | 244 | 551.0 |
| *Pelobates syriacus* | 635.83 | 1498 | 306 | 902.0 |
| *Pelobates varaldii* | 528.33 | 535 | 521 | 528.0 |
| *Leptobrachium nigrops* | 2478.40 | 2626 | 2406 | 2516.0 |
| *Megophrys nasuta* | 2588.82 | 3655 | 1853 | 2754.0 |

|  | Precip. wettest quarter (Bio16) midpoint | Precip. wettest quarter (Bio16) mean | Precip. seasonality (Bio15) midpoint | Precip. seasonality (Bio15) mean |
| --- | --- | --- | --- | --- |
| *Pelodytes caucasicus* | 261.0 | 258.50 | 34.8879 | 35.3947 |
| *Pelodytes ibericus* | 275.5 | 279.31 | 64.8089 | 68.8108 |
| *Pelodytes punctatus* | 240.5 | 186.38 | 30.8242 | 32.9111 |
| *Scaphiopus couchii* | 364.5 | 213.39 | 81.5698 | 79.9763 |
| *Scaphiopus holbrookii* | 447.0 | 395.81 | 37.2658 | 23.8257 |
| *Scaphiopus hurterii* | 268.5 | 321.63 | 63.3803 | 34.4826 |
| *Spea bombifrons* | 218.0 | 209.96 | 69.5653 | 63.1592 |
| *Spea hammondii* | 241.0 | 200.41 | 81.5272 | 89.0626 |
| *Spea intermontana* | 181.0 | 106.87 | 42.7851 | 36.7420 |
| *Spea multiplicata* | 583.5 | 274.48 | 76.4948 | 84.7081 |
| *Pelobates cultripes* | 260.0 | 183.38 | 51.0259 | 42.9586 |
| *Pelobates fuscus* | 181.0 | 203.34 | 30.7964 | 25.3717 |
| *Pelobates syriacus* | 408.5 | 301.50 | 71.7813 | 67.5895 |
| *Pelobates varaldii* | 257.5 | 257.00 | 80.1063 | 79.4594 |
| *Leptobrachium nigrops* | 837.5 | 839.50 | 21.2462 | 24.0226 |
| *Megophrys nasuta* | 1117.5 | 920.53 | 34.2204 | 29.9095 |

|  | logQ | logQ2 |
| --- | --- | --- |
| *Pelodytes caucasicus* | 2.930874 | 3.36786059 |
| *Pelodytes ibericus* | 2.680525 | 3.148966534 |
| *Pelodytes punctatus* | 2.667251 | 3.138286909 |
| *Scaphiopus couchii* | 2.385545 | 2.801818782 |
| *Scaphiopus holbrookii* | 3.078841 | 3.339258624 |
| *Scaphiopus hurterii* | 3.189597 | 3.185752681 |
| *Spea bombifrons* | 2.836357 | 2.914309326 |
| *Spea hammondii* | 2.426563 | 2.938107048 |
| *Spea intermontana* | 2.296811 | 2.859726794 |
| *Spea multiplicata* | 2.494588 | 3.106220283 |
| *Pelobates cultripes* | 2.622509 | 3.05267081 |
| *Pelobates fuscus* | 2.959468 | 3.299837472 |
| *Pelobates syriacus* | 2.772861 | 3.061405932 |
| *Pelobates varaldii* | 2.6895 | 3.072390871 |
| *Leptobrachium nigrops* | 3.651171 | 4.664670127 |
| *Megophrys nasuta* | 3.522749 | 4.70524865 |
