# Supplementary material for: Evolution of Rapid Development in Spadefoot Toads Is Unrelated to Arid Environments
Source: PLoS One. 2014 May 6;9(5):e96637. doi: 10.1371/journal.pone.0096637 (PMC4011863; doi:10.1371/journal.pone.0096637)
Supplement: Table S2 — Comparison of three evolutionary models for each climatic and life-history variable. (DOC) [file pone.0096637.s002.doc]

Table S2. Comparison of the fit of different evolutionary models to the life-history and climatic variables (BM = Brownian motion; OU = Ornstein-Uhlenbeck; lambda = estimated lambda; WN = white noise). The best-fitting model is boldfaced.

| Variable | Model | Likelihood | AIC |
| --- | --- | --- | --- |
|  |  |  |  |
| Mininum larval period | BM | -81.82293 | 167.6459 |
|  | OU | -79.43485 | 164.8697 |
|  | **lambda** | **-79.29352** | **164.587** |
|  | WN | -218.5055 | 441.0111 |
|  |  |  |  |
| Midpoint larval period | BM | -86.12684 | 176.2537 |
|  | OU | -81.75226 | 169.5045 |
|  | **lambda** | **-80.98918** | **167.9784** |
|  | WN | -274.8337 | 553.6673 |
|  |  |  |  |
| Max. larval period | BM | -95.33539 | 194.6708 |
|  | OU | -84.84019 | 175.6804 |
|  | **lambda** | **-83.45618** | **172.9124** |
|  | WN | -351.0312 | 706.0623 |
|  |  |  |  |
| Min. hatching time | BM | -28.27883 | 60.55766 |
|  | OU | -27.61654 | 61.23308 |
|  | **lambda** | **-26.70531** | **59.41062** |
|  | WN | -32.63316 | 69.26632 |
|  |  |  |  |
| Midpoint hatching time | BM | -35.63447 | 75.26894 |
|  | **OU** | **-33.27536** | **72.55072** |
|  | lambda | -33.54415 | 73.0883 |
|  | WN | -35.32057 | 74.64113 |
|  |  |  |  |
| Max. hatching time | BM | -47.39925 | 98.7985 |
|  | **OU** | **-41.91571** | **89.83141** |
|  | lambda | -42.34842 | 90.69685 |
|  | WN | -42.50687 | 89.01374 |
|  |  |  |  |
| Genome size | BM | -9.086036 | 22.17207 |
|  | OU | -9.086036 | 24.17207 |
|  | **lambda** | **-8.933632** | **23.86726** |
|  | WN | -12.91819 | 29.83637 |
|  |  |  |  |
| Min. annual precip. | BM | -1541.495 | 3086.99 |
|  | **OU** | **-119.2573** | **244.5145** |
|  | lambda | -1056.63 | 2119.261 |
|  | WN | -32753.62 | 65511.25 |
|  |  |  |  |
|  |  |  |  |
| Mean annual precip. | BM | -1450.058 | 2904.116 |
|  | **OU** | **-118.7442** | **243.4884** |
|  | lambda | -1156.774 | 2319.548 |
|  | WN | -36539.69 | 73083.37 |
|  |  |  |  |
| Max. annual precip. | BM | -2638.614 | 5281.227 |
|  | **OU** | **-123.7129** | **253.4258** |
|  | lambda | -1714.844 | 3435.689 |
|  | WN | -49803.61 | 99611.23 |
|  |  |  |  |
| Mean precip. wettest quarter | BM | -253.4193 | 510.8386 |
|  | **OU** | **-102.6168** | **211.2336** |
|  | lambda | -192.765 | 391.53 |
|  | WN | -3958.31 | 7920.62 |
|  |  |  |  |
| Mean precip. seasonality | BM | -82.25636 | 168.5127 |
|  | **OU** | **-73.04838** | **152.0968** |
|  | **lambda** | **-73.04838** | **152.0968** |
|  | WN | -94.81359 | 193.6272 |
|  |  |  |  |
| logQ | BM | -10.08336 | 24.16671 |
|  | OU | -6.808362 | 19.61672 |
|  | **lambda** | **-5.830601** | **17.6612** |
|  | WN | -6.988616 | 17.97723 |
|  |  |  |  |
| logQ2 | **BM** | **-3.689365** | **11.37873** |
|  | OU | -3.689365 | 13.37873 |
|  | lambda | -3.689365 | 13.37873 |
|  | WN | -13.12626 | 30.25251 |
|  |  |  |  |
| Larval period (specific localities) | BM | -85.25061 | 174.5012 |
|  | OU | -80.62983 | 167.2597 |
|  | **lambda** | **-79.69958** | **165.3992** |
|  | WN | -214.6818 | 433.3636 |
|  |  |  |  |
| Annual precip. (specific localities) | BM | -1971.51 | 3947.02 |
|  | **OU** | **-121.1867** | **248.3733** |
|  | lambda | -976.706 | 1959.412 |
|  | WN | -27638.39 | 55280.78 |
|  |  |  |  |
