# Supplementary material for: Evolution of Rapid Development in Spadefoot Toads Is Unrelated to Arid Environments
Source: PLoS One. 2014 May 6;9(5):e96637. doi: 10.1371/journal.pone.0096637 (PMC4011863; doi:10.1371/journal.pone.0096637)
Supplement: Table S3 — Summary of life-history and climatic data for each species using matched localities for climatic and developmental data for 12 of the 16 species (see Appendix S3). (DOC) [file pone.0096637.s003.doc]

Table S3. Summary of data for larval period and annual precipitation based on specific localities for which both are known, targeting localities with the minimum value known for larval period (see Appendix S3). Larval periods are in days, and precipitation is in mm/year. If no specific localities were associated with the reported larval period, we used the minimum larval period for the species and the minimum value for annual precipitation across the species range (for *Pelodytes caucasica, Pelobates syriacus, Pelobates varaldii, Megophrys nasuta*).

|  | Larval period | Annual precipitation |
| --- | --- | --- |
| *Pelodytes caucasicus* | 80 | 606 |
| *Pelodytes ibericus* | 70 | 527 |
| *Pelodytes punctatus* | 93 | 607 |
| *Scaphiopus couchii* | 7 | 305 |
| *Scaphiopus holbrookii* | 14 | 1294 |
| *Scaphiopus hurterii* | 13 | 907 |
| *Spea bombifrons* | 14 | 632 |
| *Spea hammondii* | 30 | 460 |
| *Spea intermontana* | 49 | 235 |
| *Spea multiplicata* | 12 | 366 |
| *Pelobates cultripes* | 93 | 421 |
| *Pelobates fuscus* | 93 | 679 |
| *Pelobates syriacus* | 70 | 306 |
| *Pelobates varaldii* | 186 | 521 |
| *Leptobrachium nigrops* | 62 | 2429 |
| *Megophrys nasuta* | 77.5 | 1853 |
