# Supplementary material for: Evolution of Rapid Development in Spadefoot Toads Is Unrelated to Arid Environments
Source: PLoS One. 2014 May 6;9(5):e96637. doi: 10.1371/journal.pone.0096637 (PMC4011863; doi:10.1371/journal.pone.0096637)
Supplement: Table S4 — Relationships between climatic and life-history variables in pelobatoid frogs using phylogenetic generalized least squares (PGLS), utilizing the OU model. (DOC) [file pone.0096637.s004.doc]

**Table S4.** Relationships between climatic and life-history variables in pelobatoid frogs using phylogenetic generalized least squares (PGLS), utilizing the OU model.

To implement the OU model, the tree was transformed using estimated values of alpha with geiger. For any given pair of variables analyzed, the tree was transformed based on the alpha value of the variable with the better fit to the OU model (see Table S2). These variables are italicized in the table below. The following variables (with estimated alpha value) were analyzed: midpoint larval period (2.105753*10^-18^), midpoint hatching time (2.229921*10^-2^), mean annual precipitation (1.397182*10^-17^), mean precipitation of wettest quarter (5.150908*10^-18^), minimum annual precipitation (1.660912*10^-17^), mean precipitation seasonality (5.618637*10^-19^), aridity (2.594917), and genome size (2.158511*10^-18^). A separate analysis was performed for the larval period and the annual precipitation for specific localities (with annual precipitation used to estimate alpha, 6.618401 *10^-18^). Significant relationships (P < 0.05) are boldfaced. Midpoint larval period and midpoint hatching times refers to the midpoint between the highest and lowest values reported for a species (Appendix S1). Minimum refers to the lowest value. For climatic variables, mean refers to the mean among localities for a species, and minimum the lowest value among localities within a species. Variables that are underlined are those for which the OU model has the best fit overall (see Table S2), and not merely the better fit for a given pair of variables. Two relationships change from significant to non-significant using the OU model (relative to the lambda; indicated with one asterisk) whereas two relationships change from non-significant to significant (two asterisks). Importantly, in all three cases, neither variable has OU as its best-fitting model (meaning that only the results from the lambda model should be considered, as in Table 1).

| Variables | R^2^ | P-value |
| --- | --- | --- |
| midpoint larval period ~ *mean annual precipitation* | 0.0253 | 0.5567 |
| midpoint larval period ~ *mean precip. wettest quarter* | 0.0081 | 0.7401 |
| midpoint larval period ~ *mean precip. seasonality* | 0.0079 | 0.7440 |
| midpoint larval period ~ *aridity (logQ)* | 0.0118 | 0.6894 |
| *midpoint hatching time* ~ mean annual precipitation | 0.1430 | 0.1537 |
| *midpoint hatching time* ~ mean precip. wettest quarter | 0.0963 | 0.2849 |
| **midpoint hatching time* ~ mean precip. seasonality | 0.1982 | 0.0735 |
| *midpoint hatching time* ~ aridity (logQ) | 0.0668 | 0.4189 |
| minimum larval period ~ *min. annual precipitation* | 0.0111 | 0.6982 |
| minimum hatching time ~ *min. annual precipitation* | 0.0012 | 0.9012 |
| larval period ~ *annual precipitation* (specific localities) | 0.0049 | 0.7971 |
| *****midpoint hatching time* ~ midpoint larval period** | **0.2644** | **0.0296** |
| **minimum hatching time* ~ minimum larval period | 0.2180 | 0.0562 |
| **midpoint larval period ~ *genome size*** | **0.5260** | **0.0300** |
| **minimum larval period ~ *genome size*** | **0.5358** | **0.0276** |
| midpoint hatching time ~ *genome size* | 0.0743 | 0.6398 |
| *minimum hatching time ~ *genome size* | 0.1603 | 0.3791 |
