# Supplementary material for: Evolution of Rapid Development in Spadefoot Toads Is Unrelated to Arid Environments
Source: PLoS One. 2014 May 6;9(5):e96637. doi: 10.1371/journal.pone.0096637 (PMC4011863; doi:10.1371/journal.pone.0096637)
Supplement: Appendix S1 — Data on life history variables in pelobatoid frogs and associated literature references. (DOC) [file pone.0096637.s005.doc]

**Appendix S1. Summary of life-history data for pelobatoid frogs.**

Summary of literature data on larval period for pelobatoid frogs (days or months). Abbreviations: *Sc. = Scaphiopus; Sp. = Spea; Pb. = Pelobates; Pd. = Pelodytes.*

| Species | Larval period (days) | Field, lab or  artificial ponds | Reference |
| --- | --- | --- | --- |
| *Sc. couchii* | 27–30 | Field | Strecker (1908) |
|  | 7–14 | Field | King (1932) |
|  | 10 | Lab | Mayhew (1965) |
|  | 13–14 | Field | Bragg (1967) |
|  | 12–16 | Artificial Ponds | Newman (1988) |
|  | 12–16 | Lab | Newman (1994b) |
|  | 18–26 | Lab | Newman (1994a) |
|  | 10.5 | Field | Morey & Reznick (2000) |
|  | 12 | Lab | Morey & Reznick (2000) |
|  | 15–40 | Field | Wright & Wright (1949) |
|  | 10.4 |  | Morey & Reznick (2004) |
| *Sc. holbrookii* | 16 or 21? | Lab | Abbott (1884) |
|  | 60 | Field | Overton (1914) |
|  | 14–28 | Field | Wright (1932) |
|  | 48–63 | Artificial Ponds | Driver (1936) |
|  | 19–22 | Lab | Ball (1936) |
|  | 26–50 | Field | Richmond (1947) |
|  | 10–56 | Lab | Gosner & Black (1955) |
|  | 27–86 | Lab | Semlitsch & Caldwell (1982) |
|  | 14–60 | Field | Wright & Wright (1949) |
| *Sc. hurterii* | 30 | Lab | Bragg (1944) |
|  | 23–51 | Lab | Blair (1949) |
|  | 21 | Field | Blair (1949) |
|  | 35 | Field | Bragg (1961) |
|  | 13–28 | Field | Bragg (1965) |
|  | 13–14 | Field | Bragg (1967) |
| *Sp. bombifrons* | 36–39 | Field | Gilmore (1924) |
|  |  | Lab | Trowbridge & Trowbridge (1937) |
|  | 32 | Lab | Trowbridge (1942) |
|  | 40 | Field | Smith (1950) |
|  |  | Field | Stebbins (1951) |
|  | 15–20 | Field | Voss (1961) |
|  | 14 | Field | Bragg (1967) |
|  | 36–40 | Field | Wright & Wright (1949) |
| *Sp. hammondii* | 51 | Lab | Burgess (1950) |
|  | 30–40 | Field | Stebbins (1951) |
|  | 28 | Lab | Denver (1993) |
|  | 57 | Field | Morezy & Reznick (2001) |
|  | 21 | Lab | Morezy & Reznick (2001) |
|  |  | Field | Wright & Wright (1949) |
| *Sp. intermontana* | 30–60 | Field | Nussbaum et al. (1983) |
|  | 36 | Lab | Brown (1989) |
|  |  | Field | Wright and Wright (1949) |
|  | 47 | Field | Morey & Reznick (2000) |
|  | 23 | Lab | Morey & Reznick (2000) |
|  |  | Lab | Hal*l et a*l. (1997) |
|  | 39–63 |  | Morey & Reznick (2004) |
| *Sp. multiplicata* | 12–16 | Field | Pomeroy (1981) |
|  | 35–40 | Lab | Woodward (1986) |
|  | 18–23 | Field | Pfenni*g et a*l. (1991) |
|  | 20–22 | Artificial Ponds | Pfenni*g et a*l. (1991) |
| *Pb. cultripes* | 3 mo. | Field | Lizan*a et al*. (1994) |
|  | 4 mo. | Field | Busack & Zug (1976) |
|  | 4 mo. | Field | Serra &Albuquerque (1963) |
|  | 3+ mo. | Field | Cei & Crespo (1971) |
|  | 36-84 | Lab | Talavera & Sanchiz (1987) |
|  | 4–6 mo. | Field | Diaz-Paniagua (1988) |
|  | 4–6 mo. |  | Arnold (2002) |
|  | 3–6 mo. |  | Garcia-Paris *et al.* (2004) |
| *Pb. fuscus* | 3–4 mo. | Field | Gislen & Kauri (1959) |
|  | 3–4 mo. or  overwinter | Field | Kuzmi*n et a*l. (1996) |
|  | 5 mo. or overwinter | Field | Kuzmin (1999) |
|  | 2–5 mo. |  | Arnold (2002) |
| *Pb. syriacus* | 3 mo. | Artificial Pool | Ugurtas (1995) |
|  | 4 mo. | Lab | Ugurtas (1995) |
|  | 70–92 or overwinter | Field | Kuzmin (1999) |
|  | 3-4 mo. |  | Arnold (2002) |
| *Pb. varaldii* | 6–8 mo. | Field | Schleic*h et a*l. (1996) |
| *Pd. punctatus* | 3–5 mo. or overwinter | Field | Toxopeu*s et a*l. (1993) |
| *Pd. punctatus* | 73 | Lab | Balcells (1955) |
|  | 3 mo. |  | Arnold (2002) |
| *Pd. ibericus* | 70 | Field | Diaz-Paniagua (1988) |
|  | 40–98 |  | Garcia-Paris *et al.* (2004) |
| *Pd. caucasicus* | 3–4 mo. |  | AmphibiaWeb |
|  | 80 |  | http://www.nafcon.dircon.co.uk/ |
| *Leptobrachium nigrops* | 2–3 mo. or longer | Field | Leong & Chou (1999) |
| *Megophrys nasuta* | 2.5–3.5 mo. | Lab | Wildenhues *et al.* (2012) |
|  |  |  |  |

Summary of literature on hatching times of pelobatoid frogs (d = days; hr = hour). Abbreviations: *Sc. = Scaphiopus; Sp. = Spea; Pb. = Pelobates; Pd. = Pelodytes.*

| Species | Hatching time | Field, Lab or  Artificial Ponds | Reference |
| --- | --- | --- | --- |
| *Sc. couchii* | 32 hr | Field | Newman (1989) |
|  | 3 d | Lab | Bucholz & Hayes (2002) |
| *Sc. holbrookii* | 1–15 d |  | AmphibiaWeb (2012) |
|  | 3 d | Lab | Bucholz & Hayes (2002) |
| *Sc. hurterii* | 3 d | Lab | Bucholz & Hayes (2002) |
| *Sp. bombifrons* | 2 d |  | AmphibiaWeb (2012) |
|  | 20 hr | Lab | Justus *et al.* (1977) |
|  | 3 d | Lab | Bucholz & Hayes (2002) |
| *Sp. hammondii* | 3–4 d |  | AmphibiaWeb (2012) |
|  | 3 d | Lab | Bucholz & Hayes (2002) |
| *Sp. intermontana* | 2-4 d |  | AmphibiaWeb (2012) |
|  | 3 d | Lab | Bucholz & Hayes (2002) |
|  | 2 d | Lab | Hall *et al.* (1997) |
| *Sp. multiplicata* | 42–48 hr |  | AmphibiaWeb (2012) |
|  | 3 d | Lab | Bucholz & Hayes (2002) |
| *Pb. cultripes* | 6 d | Lab | Bucholz & Hayes (2002) |
| *Pb. fuscus* | 5–11 d |  | AmphibiaWeb (2012) |
| *Pb. syriacus* | 7 d | Lab | Bucholz & Hayes (2002) |
| *Pb. varaldii* | 7 d | Field | Pasteur & Bons (1959) |
| *Pd. punctatus* | 4–14 d | Field | Toxopeu*s et a*l. (1993) |
| *Pd. ibericus* | 6–9 d |  | AmphibiaWeb (2012) |
| *Pd. caucasicus* | 3–4 d |  | Lukina & Koneva (1996) |
| *Megophrys nasuta* | 1 week | Lab | Wildenhue*s et al*. (2012) |

**References**

Abbott, C. C. 1884. Recent studies of the spadefoot toad. American Naturalist. 18:1075-1080.

[AmphibiaWeb](http://amphibiaweb.org/): Information on amphibian biology and conservation. [web application]. 2012. Berkeley, California: AmphibiaWeb. Available: <http://amphibiaweb.org/>.

Arnold, E.N. 2002. Reptiles and amphibians of Europe. Princeton University Press, Princeton, NJ.

Balcells, E. R. 1955. Contributions to the study of the life cycle of Spanish amphibians. British J. Herpetol. 2:1–6.

Ball, S. C. 1936. The distribution and behavior of the spadefoot toad in Connecticut. Transactions of the Connecticut Academy of Arts and Sciences. 32:351-379.

Blair, W. F. 1949. Development of the solitary spadefoot toad in Texas. Copeia 1949:72.

Bragg, A. N. 1944. Breeding habits, eggs, and tadpoles of *Scaphiopus hurterii*. Copeia 1944:230–241.

Bragg, A. N. 1961. The behavior and comparative developmental rates in nature of tadpoles of a spadefoot, a toad, and a frog. Herpetologica 17:80–84.

Bragg, A. N. 1965. Gnomes of the Night. University of Pennsylvania Press, Philadelphia, Pennsylvania, U.S.A.

Bragg, A. N. 1967. Recent studies on the spadefoot toads. Bios 38:75-84.

Brown, H. A. 1989. Tadpole development and growth of the Great Basin spadefoot toad, *Scaphiopus intermontanus*, from central Washington. Canadian Field-Naturalist 103:531–535.

Bucholz, D. R. and T. B. Hayes. 2002. Evolutionary patterns of diversity in spadefoot toad metamorphosis (Anura: Pelobatidae). Copeia 2002:180–189.

Burgess, R. C. 1950. Development of spade-foot toad larvae under laboratory conditions. Copeia. 1950:49-51.

Busack, S. D., and G. R. Zug. 1976. Observations on the tadpoles of *Pelobates cultripes* from southern Spain. Herpetologica 32:130–137.

Cei, J. M., and E. G. Crespo. 1971. Remarks on some adaptive ecological trends of *Pelobates* *cultripes* from Portugal: thermal requirement, rate of development and water regulation. Arq. Mus. Bocage, 2nd Series. 3:9–36.

Denver, R. 1993. Acceleration of anuran metamorphosis by corticotropin-releasing hormone-like peptides. General and Comparative Endocrinology 91:38–51.

Diaz-Paniagua, C. 1988. Temporal segregation in larval amphibian communities in temporary ponds at a locality in SW Spain. Amphibia-Reptilia 9:15–26.

Driver, E. C. 1936. Observations on *Scaphiopus holbrookii* (Harlan). Copeia 1932:67–69.

García París, M., A. Montori Faura, and P. Herrero Solans. 2004. Amphibia: Lissamphibia. Fauna Iberica Vol. 24. Consejo Superior de Investigaciones Cientificas, Madrid. 640 p.

Gilmore, R. J. 1924. Notes on the life history and feeding habits of the spadefoot toad of the western plains. Colorado College Publications in Science Series 13:1–12.

Gislen, T., and H. Kauri. 1959. Zoogeography of the Swedish amphibians and reptiles. Acta Vertebrata. 1:193–397.

Gosner, K. L., and I. H. Black. 1955. The effects of temperature and moisture on the reproductive cycle of *Scaphiopus h. holbrookii*. American Midland Naturalist. 54:192–203.

Hall, J. A., J. H. Larsen, and R. E. Fitzner. 1997. Postembryonic ontogeny of the spadefoot toad, *Scaphiopus intermontanus* (Anura: Pelobatidae): external morphology. Herpetological Monographs 11:124–178.

Justus, J. T., M. Sandomir, T. Urquhart, and B. O. Ewan. 1977. Developmental rates of two species of toads from the desert southwest. Copeia 1977: 592–594.

King, F. W. 1932. Herpetological records and notes from the vicinity of Tucson, Arizona, July and August, 1930. Copeia 1932:175–177.

Kuzmin, S. L. 1999. The Amphibians of the Former Soviet Union. Pensoft, Sofiya, Bulgaria.

Kuzmin, S. L., V. V. Bobrov, and E. A. Dunaev. 1996. Amphibians of Moscow Province: distribution, ecology, and conservation. Zeitschrift für Feldherpetologie 3:19-72.

Leong, T. M., and L. M. Chou. 1999. Larval diversity and development in the Singapore anura (Amphibia). Raffles Bulletin of Zooogy 47:82–137.

Lizana, M., M. Rafael, and R. Matin-Sanchez. 1994. Reproductive biology of *Pelobates cultripes* (Anura: Pelobatidae) in Central Spain. Journal of Herpetology 28:19–27.

Lukina G. P. and V. A. Koneva. 1996. Notes on the biology of the Caucasian Parsley Frog (*Pelodytes caucasicus*) in Krasnodar Region. Pp. 109-116 in Kuzmin, S. L. and C. K. Dodd (editors) Advances in Amphibian Research in the Former Soviet Union. Pensoft Publishers.

Mayhew, W. W. 1965. Adaptations of the amphibian, *Scaphiopus couchii*, to desert conditions. American Midland Naturalist. 74:95–109.

Morey, S., and D. N. Reznick. 2000 A comparative analysis of plasticity in larval development in three species of spadefoot toads. Ecology 81:1736–1749.

Morey, S., and D. N. Reznick. 2001. Effects of larval density on postmetamorphic spadefoot toads (*Spea hammondii*). Ecology 82:510–522.

Morey, S., and D. N. Reznick.  2004. The relationship between habitat permanence and larval development in California spadefoot toads: field and laboratory comparisons of developmental plasticity.  Oikos 104: 172-190.

Newman, R. A. 1988. Adaptive plasticity in development of *Scaphiopus couchii* tadpoles in desert ponds. Evolution 42:774–783.

Newman, R. A. 1989. Developmental plasticity of *Scaphiopus couchii* tadpoles in an unpredictable environment. Ecology 70:1775–1787.

Newman, R. A. 1994a. Effects of changing density and food level on metamorphosis of a desert amphibian, *Scaphiopus couchii*. Ecology 75:1085–1096.

Newman, R. A. 1994b. Genetic variation for phenotypic plasticity in the larval life history of spadefoot toads (*Scaphiopus couchii*). Evolution 48:1773–1785.

Nussbaum, R. A., E. D. Brodie, and R. M. Storm. 1983. Amphibians and reptiles of the Pacific Northwest. University Press of Idaho, Moscow.

Overton, F. 1914. Long Island Fauna and Flora 3. The frogs and toads. Museum of Brooklyn, Institute of Arts and Sciences, Science Bulletin 2:21–40.

[Pasteur, G.](http://africanamphibians.lifedesks.org/biblio/author/507), and J. [Bons.](http://africanamphibians.lifedesks.org/biblio/author/508) 1959.  [Les batraciens du Maroc](http://africanamphibians.lifedesks.org/biblio/view/2402). Travaux de l'Institut Scientifique Chérifien. Série Zoologique. Rabat. 17, xvi + 241

Pfennig, D. W., A. Mabry, and D. Orange. 1991. Environmental causes of correlation between age and size at metamorphosis in *Scaphiopus multiplicatus*. Ecology 72:2240–2248.

Pomeroy, L. V. 1981. Developmental polymorphism in the tadpoles of the spadefoot toad *Scaphiopus multiplicatus*. Ph.D. Dissertation. University of California, Riverside, California, U.S.A.

Richmond, N. D. 1947. Life history of *Scaphiopus holbrookii holbrookii* (Harlan): Part I: Larval development and behavior. Ecology 28:53–67.

Schleich, H. H., W. Kästle, and K. Kabisch. 1996. Amphibians and Reptiles of North Africa. Koeltz Scientific Publishers, Koenigstein, Germany.

Semlitsch, R. D., and J. P. Caldwell. 1982. Effects of density on growth, metamorphosis, and survivorship in tadpoles of *Scaphiopus holbrooki*. Ecology 63:905­–911.

Serra, J. A., and R. M. Albuquerque. 1963. Anfibios de Portugal. Revisita Portuguesa de Zoologicia e Biologia Geral. 4:75–227.

Smith, H. M. 1950. Handbook of amphibians and reptiles of Kansas. University of Kansas Publications, Lawrence.

Stebbins, R. C. 1951. Amphibians of Western North America. University of California Press, Berkeley, California, U.S.A.

Strecker, J. K. 1908. The reptiles and batrachians of McLennan County, Texas. Proceedings of the Biological Society of Washington. 21:69-84.

Talavera, M. R., and B. Sanchiz. 1987. Temperature dependence of larval development in *Pelobates cultripes* (Preliminary experiments). Pp. 399-402. *In:* 4th Ordinary General Meeting of the Societas Europaea Herpetologica. J. J. van Gelder, H. Strijbosch, and P. J. M. Bergers (eds.). Faculty of Sciences Nijmegen, Nijmegen.

Toxopeus, A. G., M. Ohm, and J. M. Arntzen. 1993. Reproductive biology of the parsley frog, *Pelodytes punctatus*, at the northernmost part of its range. Amphibia-Reptilia. 14:131-147.

Trowbridge, A. H., and M. S. Trowbridge. 1937. Notes on the cleavage rate of *Scaphiopus bombifrons* Cope, with additional remarks on certain aspects of its life history. American Naturalist. 71:460-480.

Trowbridge, M. S. 1942. Studies on the normal development of *Scaphiopus bombifrons* Cope. II. The later embryonic and larval periods. Transactions of the American Microscopical Society. 61:66-83.

Ugurtas, I. 1995. Turkeye'deki *Pelobates syriacus* Boettger 1889 (Anura, Pelobatidae)'un Taksonomi, Biyoloji ve Dagilisi Uzerine Arastirmalar. Turkish Journal of Zoology. 19:123-145.

Voss, W.J. 1961. Rate of larval development and metamorphosis of the spadefoot toad, *Scaphiopus bombifrons*. Southwestern Naturalist 6: 168-174.

Wildenhues M., A. Rauhaus, R. Bach, D. Karbe, K. Van der Straeten, S. T. Hartwig, and T. Ziegler. 2012. Husbandry, captive breeding, larval development and stages of the Malayan horned frog *Megophrys nasuta* (Schlegel, 1858) (Amphibia: Anura: Megophryidae). Amphibian and Reptile Conservation 5:15-28.

Woodward, B. D. 1986. Paternal effects on juvenile growth in *Scaphiopus multiplicatus* (the New Mexico Spadefoot Toad). American Naturalist 128:58–65.

Wright, A.H. 1932. Life histories of the frogs of the Okefenokee Swamp, Georgia. North American Salientia (Anura) Number 2. Macmillan Press, New York.

Wright, A. H., and A. A. Wright. 1949. Handbook of frogs and toads of the United States and Canada. Cornell Univ. Press, Ithaca, NY.
