# Supplementary material for: Evolution of Rapid Development in Spadefoot Toads Is Unrelated to Arid Environments
Source: PLoS One. 2014 May 6;9(5):e96637. doi: 10.1371/journal.pone.0096637 (PMC4011863; doi:10.1371/journal.pone.0096637)
Supplement: Appendix S2 — Time-calibrated phylogeny used in the comparative analyses in nexus/newick format. (DOC) [file pone.0096637.s006.doc]

Appendix S2. Time-calibrated phylogeny used in the comparative analyses.

((((Spea_hammondii:7.329588055417003,(Spea_bombifrons:3.492452058807896,Spea_intermontana:3.4924520588078956):3.837135996609105):18.512645677661645,Spea_multiplicata:25.842233733078643):28.36493847490538,((Scaphiopus_holbrookii:13.508071254932329,Scaphiopus_hurterii:13.508071254932329):7.499549142005085,Scaphiopus_couchii:21.007620396937412):33.19955181104662):94.22101149610626,((((Pelobates_syriacus:24.439101308564002,Pelobates_fuscus:24.439101308563988):10.617763124566707,(Pelobates_varaldii:15.207407083595378,Pelobates_cultripes:15.207407083595378):19.849457349535307):75.69545379884389,(Leptobrachium_chapaense:75.83151851761917,Megophrys_nasuta:75.83151851761917):34.920799714355354):24.10711378196092,((Pelodytes_punctatus:6.246333528329527,Pelodytes_ibericus:6.246333528329527):33.53197570064964,Pelodytes_caucasicus:39.77830922897916):95.08112278495634):13.56875169015484);
