# Supplementary material for: Evolution of Rapid Development in Spadefoot Toads Is Unrelated to Arid Environments
Source: PLoS One. 2014 May 6;9(5):e96637. doi: 10.1371/journal.pone.0096637 (PMC4011863; doi:10.1371/journal.pone.0096637)
Supplement: Appendix S3 — Data on larval period matched to specific localities. (DOC) [file pone.0096637.s007.doc]

**Appendix S3.** Localities used for analysis linking minimum reported larval periods to the climate of specific localities. In species in which the specific locality where the minimum larval period was reported is unclear, we used a known locality with the next shortest larval period. If no specific localities were associated with the reported larval period, we used the minimum larval period for the species and the minimum value for annual precipitation across the species range.

| Species | Larval period (minimum for species, days) | Specific locality | Latitude and longitude | Reference |
| --- | --- | --- | --- | --- |
| Pelodytes caucasica | 80 days | None |  | AmphibiaWeb (2012) |
| Pelodytes ibericus | 70 days | Spain: Dona Ana Biological Reserve (used park center) | 37.001  -6.419 | Diaz-Paniagua (1988) |
| Pelodytes punctatus | 93 days | France: Department Pas-de-Calais: “Dunes de Slack” S. of Ambleteuse | 50.8027  1.6094 | Toxopepus et al. (1993) |
| Scaphipous couchii | 7 days | USA: Arizona: Tucson, University Farm (= Campus Farm);  intersection of N. Campbell and E. Limberlost | 32.2848  -110.9442 | King (1932) |
| Scaphiopus holbrookii | 14 days | USA: Georgia: Okefenokee Swamp  (used northern edge for latitude and longitude) | 31.0644  -82.2713 | Wright (1932), Wright & Wright (1949) |
| Scaphiopus hurterii | 13 days | USA: Oklahoma: “central Oklahoma” (Bragg, 1967);  used 16 miles E of Norman (from Bragg, 1965) | 35.232  -97.208 | Bragg (1965, 1967) |
| Spea bombifrons | 14 days | USA: “western Oklahoma” (Bragg, 1967)  use Tipton, Oklahoma (from Bragg, 1962) | 34.5034  -99.1409 | Bragg (1962, 1967) |
| *Spea hammondii* | 30 days (but with no specific locality; Stebbins, 1951); used 57 days (Morey & Reznick, 2001) | USA: California: “Our study area was located in Riverside County, California, USA, in the western Mojave Desert.”; used Banning, California (easternmost site in Riverside County in our locality database) | 33.91  -116.84 | Morey & Reznick (2001) |
| Spea intermontana | 30 days (but with no specific locality; Stebbins 1951); used 49 days (7 weeks; Morey & Reznick 2000) | USA: California: Locality given as “Mono and Inyo Counties” of California;  we have various localities in these counties, used one that is centrally located:  5 mi W and 4 mi N Benton, Mono County | 37.88  -118.57 | Morey & Reznick (2000) |
| Spea multiplicata | 12 days (Pomeroy 1981; no specific locality but SE Arizona); used 18 days (Pfennig et al., 1991) | USA: New Mexico: area near Rodeo (used latitude and longitude of Rodeo) | 31.868  -108.980 | Pfennig et al. (1991) |
| Pelobates cultripes | 93 days (3 months) | Spain: 7 km S. of Salamanca | 40.8953  -5.6605 | Lizana et al. (1993) |
| Pelobates fuscus | 93 days (3 months) | Moscow | 55.75  37.62 | Kuzmin et al. (1996) |
| Pelobates syriacus | 70 days | None |  | Kuzmin (1999) |
| Pelobates varaldii | 186 days | None |  | Schleic*h et al*. (1996) |
| Leptobrachium nigrops | 62 days | Singapore | 1.35  103.81 | Leong & Chou (1999) |
| Megophrys nasuta | 77.5 days | None |  | Wildenhues *et al.* (2012) |

**References**

[AmphibiaWeb](http://amphibiaweb.org/): Information on amphibian biology and conservation. [web application]. 2012. Berkeley, California: AmphibiaWeb. Available: <http://amphibiaweb.org/>.

Bragg, A.N. 1962. Further study of predation and cannibalism in spadefoot tadpoles. *Herpetologica* **20:** 17–24.

Bragg, A.N. 1965. *Gnomes of the Night*. University of Pennsylvania Press, Philadelphia, Pennsylvania, U.S.A.

Bragg, A.N. 1967. Recent studies on the spadefoot toads. *Bios.* **38:** 75–84.

Diaz-Paniagua, C. 1988. Temporal segregation in larval amphibian communities in temporary ponds at a locality in SW Spain. *Amphibia-Reptilia* **9:** 15–26.

King, F.W. 1932. Herpetological records and notes from the vicinity of Tucson, Arizona, July and August, 1930. *Copeia* **1932:** 175–177.

Kuzmin, S.L. 1999. *The Amphibians of the Former Soviet Union*. Pensoft, Sofiya, Bulgaria.

Kuzmin, S.L., Bobrov, V.V. & Dunaev, E.A. 1996. Amphibians of Moscow Province: distribution, ecology, and conservation. *Zeitschrift für Feldherpetologie* **3:** 19–72.

Leong, T.M., & Chou, L.M. 1999. Larval diversity and development in the Singapore anura (Amphibia). *Raffles Bulletin of Zooogy* **47:** 82–137.

Lizana, M., Rafael, M. & Matin-Sanchez, R. 1994. Reproductive biology of *Pelobates cultripes* (Anura: Pelobatidae) in Central Spain. *J. Herpetol.* **28:** 19–27.

Morey, S., & Reznick, D.N. 2000. A comparative analysis of plasticity in larval development in three species of spadefoot toads. *Ecolog*y **81:** 1736–1749.

Morey, S., & Reznick, D.N. 2001. Effects of larval density on postmetamorphic spadefoot toads (*Spea hammondii*). *Ecology* **82**: 510–522.

Nussbaum, R.A., Brodie, E.D. & Storm, R.M. 1983. *Amphibians and Reptiles of the Pacific Northwest*. University Press of Idaho, Moscow.

Pfennig, D.W., Mabry, A. & Orange, D. 1991. Environmental causes of correlation between age and size at metamorphosis in *Scaphiopus multiplicatus*. *Ecology* **72:** 2240–2248.

Pomeroy, L.V. 1981. Developmental polymorphism in the tadpoles of the spadefoot toad *Scaphiopus multiplicatus*. Ph.D. Dissertation. University of California, Riverside, California, U.S.A.

Schleich, H.H., Kästle, W., & Kabisch, K. 1996. *Amphibians and Reptiles of North Africa*. Koeltz Scientific Publishers, Koenigstein, Germany.

Toxopeus, A.G., Ohm, & Arntzen, J.M. 1993. Reproductive biology of the parsley frog, *Pelodytes punctatus*, at the northernmost part of its range. *Amphibia-Reptilia* **14:** 131–147.

Wildenhues, M., Rauhaus, A., Bach, R., Karbe, D., Van der Straeten, K., Hartwig, S.T. & Ziegler, T. 2012. Husbandry, captive breeding, larval development and stages of the Malayan horned frog *Megophrys nasuta* (Schlegel, 1858) (Amphibia: Anura: Megophryidae). *Amphibian and Reptile Conservation* **5:** 15–28.

Wright, A.H. 1932. *Life histories of the Frogs of the Okefenokee Swamp, Georgia. North American Salientia (Anura) Number 2*. Macmillan Press, New York.

Wright, A.H. & Wright, A.A. 1949. *Handbook of Frogs and Toads of the United States and Canada.* Cornell Univ. Press, Ithaca, New York.
